# Children’s and youth mental health in all policies?: a document analysis

**DOI:** 10.1186/s12889-026-26493-3

**Published:** 2026-02-10

**Authors:** Maria Fjellfeldt, Camilla Eriksson

**Affiliations:** 1 Dalarna University, Falun, Sweden; 2 Mälardalen University, Västerås, Sweden

**Keywords:** Children and youth, Mental health, Municipal governance, Document analysis, Suicide prevention

## Abstract

**Background:**

Given the extent of mental ill health among children and youth, as well as the complexity and interconnection of determinants of mental health, effective governance, including local-level policies, is of utmost importance. This study explores how municipal governing documents address the promotion of mental health in children and youth and the prevention of mental ill health and suicide.

**Methods:**

A document analysis was conducted. The websites of Sweden’s 290 municipalities were searched for governing documents addressing mental health, mental ill health, and suicide concerning children and youth. In total, 377 documents were retrieved and analysed using content analysis.

**Results:**

The findings show that children and youth mental health was addressed in a variety of municipal governing documents, ranging from specific plans for individual schools to plans for public health and urban development. In the documents, the following patterns were found: i) specific identification of groups among children and youth; ii) accountability for the promotion of mental health and prevention of mental ill health; and iii) prevention methods. Overall, municipalities identify specific groups vulnerable to mental ill health but target “all” children and youth. The primary actor for prevention is the school, which should work with evidence-based programmes focusing on psychoeducation.

**Conclusions:**

The documents contain contradictions. Vulnerable groups were identified in the documents, but they were not specifically targeted in the measures taken. Municipalities also strive to educate children and youth in mental health despite identifying issues at a societal level.

## Introduction

Mental health is one of the most significant challenges of our time. The prevalence of mental health issues is rising, particularly among children and young people, with increasing rates observed in younger age groups. The global trend of mental ill health has been demonstrated to impact the health and development of children and young people, as well as their transition into adulthood, influencing all socio-economic groups [1, 2]. In Sweden, 13% of boys aged 10–17 had either a psychiatric diagnosis or had been prescribed psychotropic medication in 2023. The corresponding proportion among girls was 12%. Specifically, 2% of boys and 4% of girls had been prescribed antidepressant medication. Since 2010, the proportion of new cases among children (10–17 years) receiving care for mental illness has increased continuously [3]. To reverse the trend the first national long-term ten-year strategy on mental health and suicide prevention was launched in January 2025 [4, 5].

Prevention strategies extend over different levels. First, universal prevention targets everyone in the population, for example, all children and youth. The second is selective prevention, which targets specific groups or individuals. This approach is used specifically to support groups at risk. Third is indicated prevention, which targets specific individuals in complex situations [6]. For children and youth, school-based universal prevention is common to reach all children and youth. Yet, selective prevention targeting groups at risk for mental ill health is more effective than universal prevention [7]. Nevertheless, although numerous evidence-based school prevention programmes have emerged, this approach has been critiqued for its narrow focus on the individual child or young person, with insufficient consideration of structural factors such as community dynamics and public policy [8].

The concept of mental health can be understood in various ways. One approach is to consider mental well-being in terms of thriving and flourishing, and percieving satisfaction with life [9]. Another approach is to prevent individuals from being in a position where suicide is regarded as the only solution [4]. Mental ill health encompasses a range of psychiatric disorders and mental health challenges, including anxiety, distress, and sleep disturbances [5, 10]. Mental health is a broad concept; thus, determinants for mental health exist on several societal levels and are in themselves interconnected, making promotion and prevention a complex issue. Dykxhoorn et al. [11] developed a conceptual framework that incorporates a range of determinants, including individual, family, community, and structural level factors. Consequently, the model encompasses determinants from genetics, gender, and ethnicity to culture, parenting, household composition, intergenerational (dis)advantage, health and social care, social support, community safety, the welfare system, climate change, and social and cultural norms.

As illustrated by Dykxhoorn et al. [11] and Verhoog et al. [12], prevention of mental ill health requires a holistic approach to health that includes both direct (education and knowledge of mental health) and indirect (climate change, norms) measures. Health promotion actions encompass the development of effective public policies, the creation of supportive environments, and the strengthening of community action [13]. Therefore, the establishment of effective governance and social structures is of the utmost importance in the pursuit of equitable public health outcomes. Achieving this objective necessitates a multifaceted approach that includes cross-sectoral collaboration, active community participation, dissemination of information and educational resources, and integration of health considerations into all policy areas, including those traditionally outside the purview of health care.

Given the extent of mental ill health among children and youth, as well as the complexity and interconnection of determinants for mental health, the mental health and suicide challenge among children and youth must be managed as a societal issue [8]. Therefore, this study aims to explore how municipal governing documents addresses the promotion of children and youth mental health and prevention of mental ill health and suicide.

In this study, Swedish governing documents are focused. In light of the global trend concerning mental health and mental ill health among children and adolescents [1, 2], and the international recognition that health promotion initiatives include the development of effective public policies [13], and requires of a holistic approach Dykxhoorn et al. [11] and Verhoog et al. [12], advancing knowledge in this field is relevant not only to Sweden but also to other countries seeking to strengthen the promotion of mental health and the prevention of mental ill health among young people through governing strategies, including official policy documents.

## Materials and methods

### Design

A documentary analysis was applied [14, 15] including the collection and qualitative analysis of governing documents.

### Setting

In the Swedish context, the issue of promoting mental health and preventing mental ill health and suicide is addressed as interconnected, and the national strategy in this field includes work with mental health, ill health, and suicide prevention [4, 5]. The strategy on mental health and suicide prevention emphasized that responsibility for mental health and suicide prevention is shared among several actors, including municipalities. In Sweden, municipal self-government is enshrined in the constitution. This grants municipalities the authority to make independent decisions. Given that Swedish municipalities vary in size, degree of urbanization, economic capacity, population, culture, and political orientation, local self-government allows each municipality to design activities based on local conditions and the needs of citizens. Self-government also safeguards local democracy, with elected assemblies making decisions on healthcare, education, and social services in close proximity to citizens, who can thereby influence decisions and demand accountability.

Municipalities are governed by politicians directly elected by citizens. There are approximately 34,500 politicians across the 290 municipalities, 97 percent of whom serve as part-time politicians [16]. Municipal self-government [16] thus enables municipalities to respond to the national strategy [17] according to their own needs and preferences.

Municipalities are responsible for several welfare services, including social services and the education system. Consequently, targeting children and youth as a demographic group necessitates health promotion initiatives at the municipal level. Sweden has a national public health goal to create societal conditions for good health for the whole population and to close the inequality gap in one generation [18]. For children and youth specifically, the focus is on early life conditions, including child and maternal care, equitable high-quality preschool, and methods that put the child’s best interests in focus, as well as knowledge, competencies, and education, which include a good learning environment, an equitable education system, and measures taken to prevent school failure. National strategies provide overarching direction, but local strategies determine the specific municipal efforts.

While the government’s role is primarily to establish conditions that enable municipalities and other actors to work strategically and effectively, it is crucial to examine how municipalities act within their own mandates and responsibilities. It is thus of utmost importance not only to scrutinize the national strategy in this field, but also to examine local government and municipal strategies to explores how municipals address the promotion of mental health in children and youth and the prevention of mental ill health and suicide.

### Sampling and procedures

The websites of Sweden’s 290 municipalities were searched for governing documents. The search terms mental health, mental ill health, and suicide prevention were used. A variety of documents were included to capture the complexity of municipal governance and address the issue of child and youth mental health at the municipal level. Thus, municipal plans and strategies on a general level, such as master plans, social sustainability plans, and public health plans, were included. Additionally, more specific plans, such as suicide prevention plans, action plans against child poverty, library plans, and cultural and leisure strategies, were included. The search was carried out by both authors between 15th of May 2024 and 23rd of September 2024. In total, 377 different governing documents were retrieved and included. Of the included policies, the number of documents from each municipality varied between one and eight.

### Analysis

Initially, a directed content analysis [19] was performed. All documents were searched using Nvivo (version 14) for content in three themes: “mental health”, “mental ill health”, and “suicide”.

Within each of these groups, additional searches were made to find content specifically related to children and youth, using the search words “children, youth, adolescents, teenagers, pupils, parents, caregivers”. Thereafter, a summative content analysis [19] was conducted to gain an overview of the frequency of the three themes identified in the documents addressing children and youth.

All content for all three categories was coded inductively into different categories using conventional content analysis [19]. The text in “mental health” was coded first, followed by the text in mental ill health, and then the text in suicide. Both authors coded together and discussed the categories and their understanding of the text. A single-meaning unit could be placed in several categories if needed.

The codes identified in the “mental health” section were subsequently evaluated as a preliminary framework for the “mental ill health” and “suicide” segments. The initial segment of “mental ill health” underwent co-coding, while the final one and a half categories were coded by a single author (CE) in a manner consistent with the initial portion of the material. The initial coding scheme was further augmented by the addition of several categories, yet three categories emerged as particularly salient throughout the entirety of the material: identified groups, accountability for measures, and methods applied.

The two authors collaboratively reviewed all the codes in the categories Identified groups, Accountability for prevention measures, and Methods applied. The codes were then structured based on their similarities and differences to make the range of the data visible. This was achieved through manual coding using colour coding, and the findings were discussed and validated with a former school psychologist who possesses extensive expertise in children and youth mental health prevention in educational settings. In the final stage, the content in all categories was sorted and presented from universal prevention to indicated prevention [6].

### Validity

The validity strategy of *peer debriefing* [20] was employed. This strategy entails engaging selected individuals who critically review the study and pose questions to ensure that the account resonates with audiences beyond the researchers themselves. The preliminary findings were presented and discussed at a research seminar in 2025, attended by eight national and international scholars with expertise in public health, psychology, sociology, and clinical neuroscience. Through this interdisciplinary dialogue, the authors’ interpretations of the results were validated and enriched by additional perspectives that further informed the analysis.

## Results

**Fig. 1:**
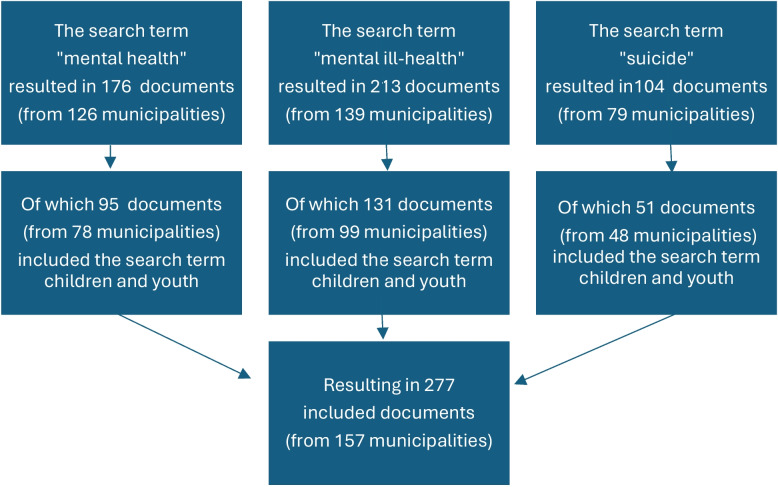
Overview of policies addressing children and youth

Children’s and youth’s mental health was addressed in a variety of municipal governing documents, ranging from specific plans for individual schools to plans for public health and urban development. In total, children and youth were addressed in 277 of the 377 included documents (see Fig. 1).

Notable differences were shown in how often municipalities address mental health, mental ill health, and suicide, respectively (See Table 1). The lowest number of occurrences was for children and youth mental health. On the contrary, for children and youth, mental ill health was most addressed in the number of policies, the occurrence of the search term, as well as in the number of municipalities. While suicide was the most frequently mentioned term for all age groups, it should be noted that these occurrences, i.e., written in the policies, do not reflect the number of municipalities or documents.

**Table 1:** Frequency of mental health, mental ill health, and suicide in all policies specifically for children and young people

Policies	**Total (n)**	**Whereof children and youth are mentioned in (n)**
Mental health (search term)		
Number of policies	176	95
Number of occurrences of the term	730	158
Number of municipalities	126	78
Mental ill health (search term)		
Number of policies	213	131
Number of occurrences of the term	790	256
Number of municipalities	139	99
Suicide (search term)		
Number of policies	104	51
Number of occurrences of the term	1228	178
Number of municipalities	79	48

Mental health in governing documents was mainly presented as mental ill health, with an emphasis on early prevention as a key priority. Factors that promote mental health are less prominent. For instance, elements such as play, physical activity, and friendship are either not mentioned or only superficially discussed. Consequently, the municipal approach to children and youth mental health can be interpreted as a preventative measure against mental ill health and suicide.

### Identified groups

The most prominent group identified in the documents was “all” children and youth, emphasising the equal rights to health for everyone from early ages to adulthood.

*“All children should have access to equal opportunities for good health and early support. The availability of early interventions needs to be strengthened. The youngest children are particularly vulnerable as they are entirely dependent on their parents for their development and well-being.”* (Gotland Action Plan for Mental Health).

There were also municipalities where no specific groups were identified. The plans were written in general terms:

*“Area: Good mental health at all ages. Target group: All residents of Essunga Municipality.”* (Essunga Public Health plan).

One specific group identified was girls and teenage girls.

*“…particularly young girls report a deterioration in mental health over time.”* (Upplands Bro Action and Activity Plan for Suicide Prevention).

Yet other identified groups were children and youth with disabilities and/or LGBTQI +.

*“The starting point is to provide good conditions for mental health in general for the entire population, but also to address the needs of particularly vulnerable target groups. Mental health issues among young people have increased, especially among young girls. Individuals with physical and mental disabilities and LGBTQI* + *persons are generally at greater risk of mental health problems.”* (Dals Ed Public Health Strategy).

Additionally, several other groups were identified in the plans. Groups identified in only a few municipalities were unaccompanied minors (Eda, Kiruna, Haparanda), specific minority groups (Göteborg), the very youngest children (Gotland), children living in vulnerable situations (i.e. children in care) (Sundsvall, Göteborg), and children living in situations characterised by violence, mental illness, and harmful use of alcohol and drugs (Göteborg, Falun). One municipality identified parents as a significant group concerning children and youths’ mental health (Götene).

In the governing documents, measures to prevent mental ill health target all children and youth. Additionally, specific groups have been identified as more vulnerable to developing mental health issues. In particular, girls, people with disabilities, and LGBTQI + people were identified, as well as children living in risky situations.

### Accountability for prevention measures

Prevention measures must be understood in relation to where the problems are attributed. Therefore, this category explores *where* the problem is described to derive from and how the municipality acts and distributes accountability and responsibility. Overall, structural factors were identified.

*“Exclusionary norms in society can severely affect children and young people who deviate from the norm.”* (Göteborg Suicide prevention plan).

However, these types of structural factors are identified as important for inequalities in mental health, but accountability lies primarily at the schools. This can be understood as it is more valuable to take measures that are in the direct environments of children and young people, instead of wider structures. This issue is addressed in various ways:

*“Structural factors such as family policy can make a difference in addressing inequalities in mental health. Schools, which reach all children and young people, play an important compensatory role in reducing mental health inequalities and improving conditions for those who are most vulnerable.”* (Gotland Action Plan for Mental Health).

The primary setting for the prevention of mental ill health and suicide is the school and school health care. School attendance is mandatory and by targeting all children and youths in schools, specific vulnerable groups are also reached. This was specifically described when all pupils were targeted in schools to reach groups considered vulnerable to mental ill health. Adults in school should educate the pupils to manage their emotions and increase life competence. School-based prevention places accountability on schools.

*“Schools can enhance their promotion of health and prevention of mental health issues by utilizing existing (evidence-based) programmes and methods, by developing equality work, and by taking action in acute situations. Children and young people should be provided with knowledge in school about signs of mental health issues through evidence-based material. Schools should have clear procedures for detecting the risk of suicide and suicide attempts.”* (Norsjö S Suicide Plan).

Education was also identified as central to mental health and “a good life”. Hence, providing good and equal education is a wider goal but also directed towards, and a part of, the mission of preventing mental ill health among children and youth.

*“Completing studies is an important factor for children and young people’s opportunities to live a good life and reduces the risk of, for example, mental health issues and social exclusion.”* (Dals Ed Public Health Strategy).

Municipalities mention that schools can also be the cause of mental ill health, which itself must be prevented. However, school-based prevention is complex and also includes the school environment and context in a wider sense. In contrast to the prevention approach, this encompasses promotion by strengthening positive aspects in schools.

*“Primary school students should be in a safe environment that strengthens self-esteem and provides hope for the future. Active efforts and enhanced student health services should prevent and counteract discrimination, bullying, and mental health issues.”* (Huddinge The Elementary School’s Operational Plan).

Schools are commonly mentioned as key actors accountable for mental health intervention to increase children’s and youths’ knowledge about mental health. Although teachers and school health care are most frequently mentioned and identified as important, *all* adults in the school setting, such as janitors, librarians, social pedagogues, special educators, pupil assistants, and school social workers, are identified as important.

*“This can include everyone from teaching assistants, school librarians, and janitors to teachers. With more adults in the school, more people can pick up on early signs of mental health issues, thereby providing more opportunities for students to receive timely help.”* (Karlskrona Municipal Budget -Strategic Plan with Financial Conditions).

Moreover, adults in school are identified as important and are accountable for supporting children and youth. Therefore, adults in school also need suicide prevention education.

*“It is important to provide early support to children and young people by strengthening pupil health services and school staff in suicide prevention work.”* (Finspång Strategy for Suicide Prevention).

Furthermore, schools are responsible for educating pupils about mental health and are also accountable for identifying suicidal behaviours. This also makes children accountable for their own mental health.

*“This can be achieved by educating children and young people about mental health, through suicide prevention and evidence-based programmes, as well as increasing awareness of mental health interventions”* (Lund Programme for Social Sustainability).

The most common approach to educating children and youth about mental health aims to increase individual knowledge and provide psychoeducation. Schools are accountable for delivering the educational programme, but the child or youth is accountable for managing their own mental health.

*“The course aims to provide young people with tools to manage difficult thoughts, feelings, and experiences.”* (Södertälje Action plan for suicide prevention).

However, for children at risk, such as those in care or from minority groups, accountability lies outside the child. Important adults should be aware of and support these children.

Despite acknowledging that upbringing, parenting, and living conditions are important, schools are identified as primarily accountable for mental health prevention measures for children and youth. Additionally, governing documents explicitly state that schools are important as a health promotion arena for enhancing knowledge about mental health. These documents also target school health care, teachers, and “all adults” within the school context.

### Methods applied

Municipalities have stated various methods to promote mental health, and prevent mental ill health and suicide, ranging from universal interventions to selective and indicated interventions [6]. These methods target both children and youth in general, as well as particular risk groups, such as children who are next of kin to someone who committed suicide.

To promote the mental health of children and youth, some general methods were identified, such as contributing to meaningful leisure activities and fostering relationships with adults. Additionally, collaboration between stakeholders, such as schools, social services, police, and other key stakeholders in civil society, was addressed in the documents. This is illustrated in action plans for youth centres:

*These activities convey hope for the future, promote personal development, and contribute to meaningful and stimulating leisure time. They promote a positive lifestyle and good health. Special attention is given to children and young people living in socially vulnerable situations. […] Close cooperation takes place with guardians, schools, social services, police, other municipal and private activities, and civil society. This enables joint co-creation and collaboration.* (Västerås Action Plan for Activities in Youth Centres and Youth Clubs).

There were also methods specifically addressed to parents and caregivers, as well as to the municipality, which should provide activities for youths not engaged in school or work.

*The municipality should invest in parental education, provide support to homes in need, develop and ensure equal student health, and establish a family centre. A well-functioning municipal activity responsible for young people is of utmost importance to quickly help those who, for some reason, neither study nor work.* (Vansbro Strategic Plan).

Efforts to address mental ill health and suicide prevention were often closely linked. The focus here was on promoting living conditions and lifestyle habits that support mental health, which can serve as protective factors throughout life.

*Universal suicide prevention focuses on people’s living conditions and promoting good life opportunities for less advantaged groups. Children and young people are one of several important target groups for universal interventions, which ideally also provide protective effects throughout life. An example is school-based programmes to strengthen protective factors and reduce risk factors.* (Mjölby Action Plan Suicide Prevention).

There were a few examples of suicide prevention efforts, such as restricting access to substances and implementing physical interventions like bridge railings and railings by train tracks. Overall, it was stated that interventions should be based on scientific knowledge and evidence. However, in suicide prevention, methods did not specifically target children and youth as primary actors, but instead as next of kin, making them vulnerable to mental ill health.

*Substance abusers, survivors of suicide, and relatives/close ones with a special focus on the children of individuals who have committed suicide should always be offered professional support.* (Göteborg Suicide Prevention Plan).

In addition, there were examples of professional structures for prevention and collaboration to support the children and youth who most need it.

Moreover, child impact assessments after suicide attempts were described as a method to prevent mental ill health and suicide.

*“A child impact assessment should always be carried out in these incident analyses for suicides and suicide attempts so that children and young people are given special attention and interventions to prevent further increased risk of suicide, suicide attempts, or suicidal actions.* (Eskilstuna Plan for suicide prevention).

In the school setting, various evidence-based methods were used among adults to engage with children and youth:

*School nurses have various methods to be able to address and see the whole individual. This includes different tools to meet the student’s needs, such as MI (Motivational Interviewing), TMO (Trauma-Informed Care),* (Gnesta School Plan Against Discrimination and Abusive Treatment).

Additionally, adults in schools were educated in methods to detect early signs and support the mental health of children and youth:

*The purpose of the course is to prevent and early detect mental health issues, bullying, and ultimately suicide among pupils, as well as teach them to handle crises, anxiety, depression, and suicidal thoughts, thereby increasing their self-esteem. The training lasts for one and a half days.* (Södertälje Action plan for suicide prevention).

However, the most prominent methods to prevent mental ill health and suicide among children and youths involve applying interventions directly towards them such as individual health conversations with the school nurse, increasing participation, and applying a norm-critical perspective in prevention.

*There is a continued need, through assignments in the action plan, to maintain focus on developing work in schools to increase pupils’ knowledge of mental health, norms and values.* (Gotland Suicide prevention plan).

In addition, municipalities mentioned specific evidence- and research-based programmes, for school prevention aimed at educating children and youth: Youth Awareness of Mental Health (YAM), Acceptance and Commitment Therapy (ACT), Dancing without Demands.

In summary, the results showed that many municipal policies identified all children and youth, although LGBTQI + individuals, girls, and children and youth with disabilities as specifically vulnerable to mental ill health. Additionally, children and youth living in vulnerable situations were addressed. Accountability for the mental health of children and youth at the societal level includes family policy (protective factors), norm formation, social exclusion, the home (early life conditions), and preschool. Above all, schools were identified as the primary actors in promoting mental health and preventing mental ill health among pupils. Within schools, accountability is further allocated to individuals: principals, teachers, janitors, and all other adults in the specific setting are addressed. Furthermore, pupils were pinpointed as accountable for their own mental health.

Methods used include collaboration among welfare agents at a structural level along with parents, schools, and social care services. Another method includes strengthening parenthood, leisure time, and living conditions (such as substance and alcohol use and level of education), as well as providing specialised social and care support to children and youth who are next of kin to a person who has committed suicide or who have themselves attempted suicide. Moreover, school nurses provide individual health conversations, and teachers conduct psychoeducation by applying evidence-based school programmes in their classes, such as YAM. Teachers should also work with a norm-critical perspective. Ultimately, the purpose of these methods is to educate pupils, as individuals, about mental health, mental ill health, and suicide prevention.

## Discussion

There are major contradictions in the governing documents. Firstly, there are contradictions within the category of accountability. While addressing structural level factors for mental ill health, such as norms, social exclusion, and early life conditions, schools are described as accountable for suicide prevention programmes and prevention of mental ill health and frequently address children and youth at the individual level. An illustrative example is norms as a societal structure being addressed in documents, but in practice, placing accountability at schools, to educate children and youth about norms, rather than actively striving to change norms at the societal level. Despite the difficulty of changing norms at a societal level, there are successful examples. One example is passive smoking, where actions were taken to promote public health on a societal level by prohibiting smoking in public places (2018:2088) instead of individualising the issue, and norms aligning with this decision followed. Another example is the Planning and Building Act (SFS, 2010:900), which states that buildings must be accessible for persons with disabilities, rather than making it an individual problem to access the building. Still, in the area of children’s and youth’s mental health, much responsibility and the solution are presented as individual issues. Although some school-based programs for mental health have a *moderate* effect [21], they are also strongly criticized for the narrow focus on the individual child [8]. An individual-focused approach risks transferring greater individual responsibility for suicide prevention onto children and adolescents, and by extension, impose a sense of guilt. Previous research demonstrates associations between delusions, sense of guilt, and suicidal ideation, tendencies, and attempts among young people [22, 23]. In children and adolescents with bipolar I disorder, delusions of guilt are uniquely associated with suicidal ideation [24]. The association also appears gendered: girls were significantly more likely than boys to attempt suicide due to feelings of guilt [23]. An individual-focused approach to educating children and adolescents in suicide prevention could thus risk unintended consequences. Evidence-based interventions with a *strong* effect: means restriction [25–27] were notably absent. This raises the question of whether municipal suicide prevention strategies differ when aimed at children and young people compared to adults. Is the restriction of means considered relevant for adults, but not for children and adolescents?

It is evident that contextual factors such as having access to a school-based health centre, region, and area of residence, and socio-economic status are positively associated with children’s and adolescents’ mental health [12]. Yet, these aspects are not mentioned. Moreover, it is notable that methods focusing on educating individuals can be understood as contradictory to the national public health objectives [18], which aim at schools as structures to promote health through a good learning environment. Avoiding this disruption in national governance requires strategic alignment of governing documents at both national and local levels, as well as internal coherence within those documents, to effectively manage mental health as a complex phenomenon. Although this study focuses on municipal-level prevention and promotion of mental health, Carbonell et al. [28] similarly argue that child and adolescent mental health care is shaped by a predominantly biomedical view, as well as by discrimination and the minimization of mental distress during childhood and adolescence. Consequently, systemic and structural barriers further hinder access to child and adolescent mental health services.

Moreover, using the school as the primary arena and educating pupils on mental health and suicide prevention means additional time spent in school with a focus on mental health education. This time could instead be used for mental health efforts, such as physical activity or cultural activities. While integrating mental health into school curricula is one of several actions proposed by the WHO’s Mental Health Action Plan [29], it is part of a broader emphasis on societal structures and governance. However, structural initiatives at the national level are taken to enable meaningful leisure time that promotes social relations and contexts for children and youth, with a special focus on targeting those in low socio-economic positions [17]. In summary, some structures might be difficult to target at the municipal level. Therefore, national and municipal forces must work together to combat complex issues such as children and youth mental health, balancing the responsibility of society, welfare systems, and the individual.

Another contradiction concerns the identified groups and the methods applied. Despite identifying specifically vulnerable groups, the methods generally reached all children and youth attending school. Hence, even though vulnerable groups were identified, few efforts were made to combat these specific risk factors at a structural level, and surprisingly few methods aimed to prevent mental ill health in these specific groups. This is notable as selective prevention has been shown to be more effective than universal prevention [7]. Also, as insufficient measures were taken in practice to strengthen prerequisites for mental health for the identified vulnerable groups, the governing documents were not aligned with the overall public health objective [18] to close the inequity gap in one generation or with the WHO action plan for mental health [29].

Additionally, this could be understood in relation to research showing that children in vulnerable situations related to sexual and/or physical abuse are not sufficiently protected and supported, as a family-oriented approach is prioritised over child security in terms of individual needs, rights, and mental health [30, 31]. Moreover, while a school-setting approach reaches the majority of children and youth, the risk for mental ill health is overrepresented in groups of children and youth with non-attendance in school [32]. Thus, those pupils who need the support and interventions the most are not reached with universal prevention. Although there might be other interventions targeting this group due to non-attendance in school and possibly other problems, the societal support for mental health among this group is not visible in the included governing documents. Consequently, children and youth who do not attend school miss out on their education, as well as health promotion and preventative interventions. Understanding school as a health-promoting structure or arena is essential, as several health determinants are specifically targeted to promote mental health in both the short-term and long-term perspectives.

The results of the summative analysis (see Table 1) can be viewed through the lens of adultcentrism [33]. This concept serves as a tool for understanding power relations and how the peripheral social positions occupied by young people generate mechanisms of distancing and a sense of marginality. The frequency with which children and youth are addressed in the studied documents risks reinforcing the perception that policy lacks a youth perspective. As noted in another study [34], this may lead young people to conclude that institutions are incapable of effecting meaningful change in their situations and lives. Consequently, young people may turn to informal and non-institutional settings to address their needs and demands. Additionally, the absence of children’s and young people’s voices is not merely symbolic,it has tangible consequences for the effectiveness of preventive policies. In a future study, it would be valuable to further examine how these policy documents implicitly construct an “ideal preventive subject” (cf. [35] as autonomous, informed, and self-regulating—a model that does not necessarily correspond to the lived realities of young people in vulnerable contexts.

Further, it is evident that governing documents addressing mental ill-health frame children as future adults rather than recognising them primarily as children [36, 37]. The analysis of the documents revealed a strong emphasis on the importance of education and knowledge as solutions to the problem at various levels. It is argued that education prevents mental ill health, as passing grades correlate with better mental health and also serve as a ticket to becoming successful in adult working life, which is argued to be a condition for mental health and well-being in adults. Having an income to earn a living as an adult is beneficial for the individual but also favours society as a welfare state financed by taxes. Although education is an important aspect of health promotion, there is a lack of recognition of the importance of shelter, food, income, a stable ecosystem, sustainable resources, social justice, and equity [13]. To combat mental ill health as a public health challenge, all levels of determinants – individual, family, community, and structural – must be addressed [11, 12]. However, this is lacking in municipal governance, and the methods overall do not align with how the problem is described. An important implication is therefore to intentionally formulate governing documents so that they align with the proposed solutions, actions, and the issues being addressed, of which ill mental health is a consequence. This specifically applies to the need to harmonise target groups and actions taken. A broader implication is that municipalities should initiate strategic efforts to address the more complex and structural factors they have identified as contributing to mental health risks among children and youth. Moreover, municipalities could address some of these issues by increasing collaboration with academia to improve their governing documents, as also suggested by Carbonell et al. [28]. Finally, policy documents need to be child-centered [38] to convince children and young people that institutions and societal actors working to promote their mental health possess the ability to make change in their current life situations.

### Limitations and Strengths of the Study

One limitation of the study is that the researchers collected publicly available documents from municipal websites, which implies that additional relevant documents may have existed but were inaccessible to the research team. Due to practical constraints, the researchers were unable to contact officials in the 290 included municipalities. The researchers assessed that the collected material—comprising 377 documents, of which 277 addressed children and youth—was sufficient to conduct the study.

Another limitation concerns the data analysis approach: instead of reading all documents in their entirety, the researchers employed specific search terms within the software Nvivo (version 14). This means that municipalities may have used alternative terminology when describing their work related to child and adolescent mental health and suicide prevention, which may not have been captured by the selected search terms. To ensure broad coverage of the topic, a wide range of keywords was used, including “children,” “youth,” “adolescents,” “teenagers,” “pupils,” “parents,” and “caregivers.”

A third limitation relates to the exclusive focus on municipal documents. As a result, the study did not include the health care sector’s work on mental illness, including self-harming, waiting lists and system fragmentation. While self-harm is a key indicator of psychological distress and suicide risk among younger populations, and is closely associated with suicide prevention, this area falls outside the scope of the present study.

One strength of the study is that its preliminary findings were reviewed and discussed during a research seminar in 2025, attended by eight national and international researchers specializing in public health, psychology, sociology, and clinical neuroscience. The authors’ interpretations of the results were validated through this interdisciplinary dialogue, which also contributed additional perspectives to the analysis.

## Conclusions

The study showed that children’s and youth’s mental health was addressed in a variety of municipal governing documents, ranging from specific plans for individual schools to plans for public health and urban development. A pattern emerged of municipalities striving to educate children and youth, both generally and specifically on mental health, through evidence-based suicide prevention programmes. This can be understood as schools, children, and youths being made accountable for mental health measures. While *all* children and young people are identified as vulnerable due to their age, they are also responsible for acting to promote their own mental health and prevent mental ill health. Specific groups were identified in the documents but not specifically targeted in the measures taken. Instead, universal prevention was applied, with the risk of not reaching those children and youth who need it the most. This could be considered a reactive solution at the individual and school level, where long-term societal solutions to decreasing mental ill health were absent. In the long-term, structural determinants of mental health, such as climate change, commercial factors, economic conditions, and the design of the welfare system, must be managed on a national level, as this is at least partly beyond the municipal responsibility and mandate.

## Data Availability

All documents included are available on the municipalities' websites.
